# Methylenetetrahydrofolate dehydrogenase (MTHFD) enzyme polymorphism as a maternal risk factor for trisomy 21: a clinical study 

**Published:** 2010-11-25

**Authors:** D Neagos, R Cretu, A Tutulan–Cunita, V Stoian, LC Bohiltea

**Affiliations:** * ‘Carol Davila’ University of Medicine and Pharmacy, Department of Genetics, Bucharest Romania; ** Medical Genetics Laboratory, ‘Victor Babes’ National Institute of Pathology, Bucharest Romania; *** Department of Genetics, Faculty of Biology, University of Bucharest Romania

**Keywords:** Down syndrome, folate, methylenetetrahydrofolate dehydrogenase, (MTHFD), MTHFD1 1958G>A

## Abstract

Recent reports linking Down syndrome (DS) to maternal polymorphism at the methylenetetrahydrofolate dehydrogenase (MTHFD) locus have generated a great interest among investigators in the field. In the current study, we examine one genetic polymorphism involved in homocysteine/folate pathway as a risk factor for DS in a Romanian urban–area women cohort. Our results show that the frequencies of *MTHFD1* alleles, as well as the frequencies of MTHFD11958 genotypes (GG, GA, AA, GA+AA) do not correlate with DS pregnancies, demonstrating no difference between the case and control groups, as opposed to the findings of Scala et al. (2006) on an Italian cohort.

## Introduction

Down syndrome (DS) is a genetic disease resulting from the presence and expression of three copies of the genes located on chromosome 21. In 95% of DS cases, the nondisjunction of chromosome 21 occurs during meiosis I in the maturing oocyte; although advanced maternal age represents the major risk factor for DS, most children with this complex metabolic disease are born to young mothers (less than 35 years) [1]. The mechanism underlying the meiotic nondisjunction is poorly understood and is thought to have a multifactorial aetiology, being influenced by both genetic and acquired factors [2,3]. 

Abnormal folate metabolism and common folate–metabolizing enzyme variants have been described as possible risk factors for DS [2,4]. A deficiency in cellular folates (members of the B9 vitamins family) and methyl donors may be associated with abnormal DNA methylation, DNA strand breaks, defective chromosome recombination, and abnormal chromosome segregation [5,6,7,8]. The folate cycle is involved in two essential physiological processes: the synthesis of purines and pyrimidines required for DNA synthesis and repair; and the methylation associated with the methionine cycle. Lower levels of folate have been associated with increased risks of trisomy 21 and therefore, folic acid supplemental intake is now commonly recommended during pre– and post–conception and early pregnancy.

Methylenetetrahydrofolate dehydrogenase, MTHFD is an enzyme involved in folate metabolism, a trifunctional protein that mediates the interconversion of 5,10–MTHF, 5,10–methenylTHF, and 10–formylTHF. The last two are the donor cofactors for de novo purine and pyrimidine biosynthesis and, thus, for the biosynthesis of DNA [9].

**Figure 1:**
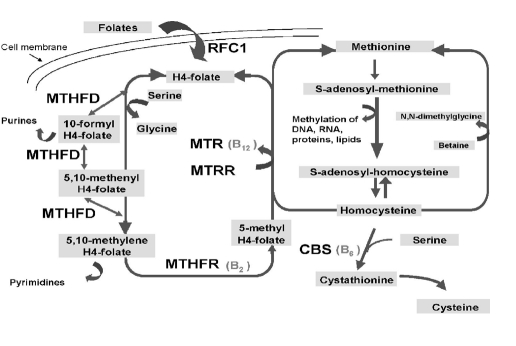
The homocysteine and folate metabolic pathway

The MTHFD1 gene is located on chromosome 14 (14q24). A common MTHFD1 1958G>A polymorphism (Arg653Gln) reduces the enzyme's activity and stability and has been associated with an increased risk of several human diseases, including neural tube defects, congenital heart defects and unexplained second semester pregnancy loss [10,12,12].

Our paper addresses the relationship between MTHFD1 1958G>A polymorphism and the risk of DS pregnancy in a Romanian urban-area cohort of women.

## Materials and methods

Our study included 26 women who gave birth to DS babies and 46 control mothers of healthy children. The case mothers were interviewed and a questionnaire regarding age, place of residency (rural/urban) during the pregnancy period, education, obstetric history, and periconceptional folic acid intake was completed. The DS mothers were younger than 40 years old and 7 of them had a history of spontaneous miscarriages (26.92%). In all cases, their babies were confirmed of having classic trisomy 21. The control blood samples were collected from 46 women who gave birth only to healthy children, without any history of miscarriages or abnormal pregnancies. An informed consent was obtained from all participants. All women in our study reside in the same geographic area and have a similar social background. 

Peripheral blood samples (5 ml) were collected on EDTA from all participants. Genomic DNA was isolated from the whole blood by using PeqGOLD Blood DNA mini kit, while following the manufacturer's instructions. 

The presence of MTHFD1 1958G>A mutation was analyzed by polymerase chain reaction followed by a restriction fragment length polymorphism analysis–allele specific restriction digestion with MspI (PCR–RFLP). Primers for amplification were forward: 5'–CCTGGTTTCCACAGGGCACTC–3' and reverse 5'–CCACGTGGGGGCAGAG GCCGGAATACCGG–3'. PCR conditions were denaturation 94 degrees C (1 minute), annealing 60 degrees C (1 minute), elongation 72 degrees C (1minute) for a total of 36 cycles. The PCR amplification generated a 310 bp fragment. The transition observed in MTHFD1 1958G>A variant abolishes a MspI restriction site; therefore after MspI digestion of the PCR product, the A–allele was detected by the presence of 282 and 28 bp fragments, while the G–allele was identified by the presence of 196, 86 and 28 bp fragments.

### Statistical Analysis

**Table 1:** Allele frequencies of MTHFD 1958 G>A in mothers of DS children and control mothers.

Genotype	Allele	DS mothers (%)	Control mothers (%)	Chi Test(X^2^)	p value
1958	G	29(55.76%)	55(59.78%)	0.22	0.63
	A	23(44.23%)	37(40.22%)		
	Total	52	92		

Allele frequencies were calculated for each genotype, and the differences in allele frequencies between mothers of children with DS and control mothers were determined by using a chi–square test. Expected genotype frequencies were calculated from the allele frequencies under the assumption of Hardy–Weinberg equilibrium. The interaction between the two MTHFD1 genotypes was evaluated by calculating the odds ratios and p values for mutant genotypes. The analyses were performed while using the SPSS software

## Results and discussions

**Figure 2:**
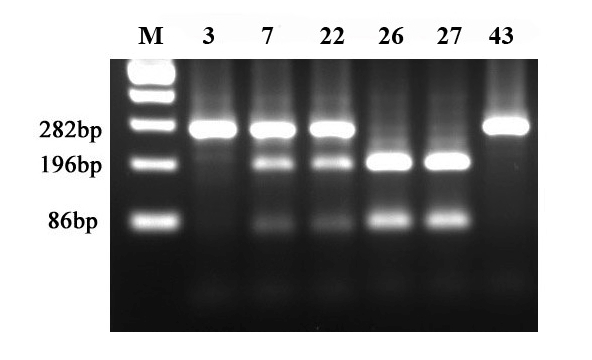
PCR–RFLP (Msp1) mutational analysis of MTHFD 1958G>A polymorphism. Lane 1–case 3; 2–case 7; 3–case 22 ; 4–case 26; 5–case 27; 6–case 43; M–pGEM 100 bp molecular weight marker (Promega).

In this study, we examined one polymorphism in methylenetetrahydrofolate dehydrogenase (MTHFD1)–gene encoding a folate–metabolizing enzyme–as a maternal risk factor for meiotic nondisjunction of chromosomes 21, causing DS, in a cohort of Romanian mothers. The investigation of the polymorphism was addressed by PCR amplification of genomic DNA by using the primers described in Materials and Methods section, followed by restriction digestion with an appropriate endonuclease. The results of the mutational analysis are shown for a few representative cases in the (Figure 2).

As observed in Figure 2, following PCR–RFLP analysis, cases 3 and 43 have the homozygous 1958AA genotype, cases 26 and 27–homozygous 1958GG genotype and cases 7 and 22–heterozygous 1958 AA genotype.

The allele frequencies of MTHFD1 1958G>A in DS mothers and control mothers are listed in Table 2. MTHFD1 1958 A allele frequency was 44.23% in DS mothers (X^2^: 0.22, p value 0.63).

**Table 2:** Allele frequencies of MTHFD 1958 G>A in mothers of DS children and control mothers

Genotype	DS mothers (%)	Control mothers (%)	Odds ratio	95% CI	p value
GG	6 (23.07%)	15 (32.60%)	1	Reference	
GA	17 (65.38%)	25 (54.34%)	1.7	0.54–5.26	0.35
AA	3 (11.53%)	6 (13.04%)	1.25	0.23–6.69	0.79
GA or AA	20 (76.92%)	31 (67.39%)	1.61	0.53–4.85	0.39

As far as genotypic frequencies are concerned, GA heterozygous genotype frequencies of MTHFD1 at position 1958 were higher among DS mothers than among controls mothers (65.38% versus 54.34% with an odds ratio of 1.7 (95% confidence interval (CI) 0.54–5.26) indicating that this polymorphism may have a genetic impact upon the risk of DS. AA homozygous genotype frequency at this position was higher in control mothers than in DS mothers (13.04% versus 11.53% respectively, with an odds ratio of 1.25 (95% CI 0.23 to 6.69), although not at a statistically significant level. 

Several studies performed in the last decade suggest a possible contribution of an impaired folate metabolism to MTHFD1 DS risk, and the current opinion is that the presence of mutant alleles in the genome might affect the risk of the disease. However, the studies performed so far, have often provided conflicting results [13,14,15,16] and the question is still unsolved. Certainly, trisomy 21 is the result of the interplay of several factors of genetic, epigenetic, environmental and stochastic origin; in this context all the studies performed so far [13,14,15,16] have provided some putative DS risk factors.

Scala et al. investigated the possible contribution of the MTHFD1 1958G>A polymorphism as a maternal risk factor for having a DS child and observed positive interactions for the combined MTHFD1 1958AA/RFC1 80GG genotype [17]. We investigated the prevalence of the MTHFD1 genotype variation in Romania, in our study, finding the frequencies of the GG, GA and AA genotypes at 1958 position among DS mothers of 23.07%, 65.38%, and 11.53%, respectively, and among control mothers–of 32.60%, 54.34%, and 13.04%, respectively. These data do not point to any association between the polymorphisms as this locus and the risk of having DS infants, in contrast with the previous study.

##  Conclusions

We did not find any statistically significant association between MTHFD1 polymorphic genotype and the history of DS pregnancies; thus, the relationship between MTHFD1 polymorphism and DS appears to be only a supposition and the next step in our study is the catamnestic evaluation of our patients with DS babies for two years. Additional studies are essential to unravel the complex relationship between genes, micronutrients, and folate/methyl metabolism.
